# Hepatitis C virus infection in the immunocompromised host: a complex scenario with variable clinical impact

**DOI:** 10.1186/1479-5876-10-158

**Published:** 2012-08-03

**Authors:** Anna Linda Zignego, Carlo Giannini, Laura Gragnani, Alessia Piluso, Elisa Fognani

**Affiliations:** 1 Center for Systemic Manifestations of Hepatitis Viruses (MASVE), Department of Internal Medicine, University of Florence, Largo Brambilla 3, 50134, Florence, Italy; 2 Istituto Toscano Tumori (ITT), Florence, Italy

**Keywords:** Hepatitis C Virus (HCV), Hepatic and extrahepatic disorders, Immunopathogenesis of HCV-related damage, Immunosuppression, Hypogammaglobulinemia, Bone marrow transplantation, Liver transplantation, Kidney transplantation, HCV-HIV coinfection, Liver fibrosis progression

## Abstract

The relationship between Hepatitis C Virus (HCV) infection and immunosuppression is complex and multifaceted. Although HCV-related hepatocytolysis is classically interpreted as secondary to the attack by cytotoxic T lymphocytes against infected cells, the liver disease is usually exacerbated and more rapidly evolutive in immunosuppressed patients. This generally occurs during the immunosuppression state, and not at the reconstitution of the host response after immunosuppressive therapy discontinuation. The field of immunosuppression and HCV infection is complicated both by the different outcome observed in different situations and/or by contrasting data obtained in the same conditions, with several still unanswered questions, such as the opportunity to modify treatment schedules in the setting of post-transplant follow-up. The complexity of this field is further complicated by the intrinsic tendency of HCV infection in itself to lead to disorders of the immune system. This review will briefly outline the current knowledge about the pathogenesis of both hepatic and extrahepatic HCV-related disorders and the principal available data concerning HCV infection in a condition of impairment of the immune system. Attention will be especially focused on some conditions - liver or kidney transplantation, the use of biologic drugs and cancer chemotherapy - for which more abundant and interesting data exist.

## Introduction

The relationship between Hepatitis C Virus (HCV) infection and immunosuppression, when compared with Hepatitis B Virus (HBV) infection, seems quite peculiar. This is possibly secondary to the differences - both in structure and replication mechanisms as well as in the natural history of infection - existing between the viruses.

Although HCV-related hepatocytolysis is classically interpreted as secondary to the attack by cytotoxic T lymphocytes against infected cells, the liver disease is usually exacerbated and more rapidly evolutive in immunosuppressed patients. This occurs during the immunosuppression state, and not at the reconstitution of the host response after therapy discontinuation. In fact, when we compare the average time and range of years necessary for the establishment of end-stage chronic liver disease (CLD) under normal conditions and in various categories of immunocompromised patients (i.e., transplanted patients, HIV-coinfected subjects, patients with hypogammaglobulinemia), a clear difference appears, with time intervals ranging from an average period of 30 years necessary to join the end-stage CLD in normal conditions to an average interval of 2 years after liver transplantation (LT) [1] (Table 1).

**Table 1:** Time to develop liver cirrhosis in immunocompetent and immunosuppressed patients

**Cathegory of Patients**	**Median/range(years)**
**Immunocompetent**[2,3]	**30/**13-42
**Hypogammaglobulinemia**[4-6]	**8.8**/4.5-15
**HIV-HCV co-infection**[7-9]	**10**/6-30
**Bone marrow transplantation**[10,11]	**10.1**/1.2-24.9
**Liver transplantation**[12-14]	**2/**1-4

Overall, the varying behavior of the infection in different forms of immunosuppression outlines the important differences between the physiopathology of HCV- or HBV-related disorders, emphasizing the opportunities for different approaches and, not least, encouraging a deeper analysis of pathogenetic mechanisms of virus-related liver damage.

The field of immunosuppression and HCV infection is complicated both by the different behavior observed in different situations and/or by contrasting data obtained in the same conditions, with several still unanswered questions, such as the opportunity to modify treatment schedules in the setting of post-transplant follow-up. The complexity of this field is further complicated by the intrinsic tendency of HCV infection in itself to lead to disorders of the immune system. Of the great variety of situations leading to immunosuppression in the presence of HCV infection, the most numerous and interesting data derive from three conditions which are often interlinked: liver or kidney transplantation, the use of biologic drugs (monoclonal antibodies and interleukins with immunosuppressive activity) and cancer chemotherapy. The complex relationship between HCV and HIV has been previously deeply developed by others (for review see [15]) and it will not be the object of the present review.

### HCV: the burden of chronic infection and mechanisms of liver disease

#### HCV infection is a critical public health problem

There are about 200 million HCV carriers worldwide, more than 100,000 deaths every year are attributable to HCV and it is estimated that this number will significantly increase in the future [16]. HCV infection has a high propensity to persist in the host, in fact, acute infected patients fail to eradicate the virus in about 80% of cases and subsequently develop chronic infection. This condition leads to both extrahepatic and hepatic disorders, mainly chronic liver inflammation, cirrhosis and liver cancer [17]. To persist in the host, HCV uses different strategies aimed at subverting both the innate and adaptive immune responses. The immune system, in an attempt to clear the virus, induces continuous and extensive cytolytic activity on infected hepatocytes resulting in chronic inflammation, possibly evolving to severe liver disorders. The immune-mediated damage, although considered the main mechanism for HCV-related liver injury, is not exclusive, and a direct viral cytopathic effect has been suggested on the basis of experimental data (see below).

#### Innate immunity

Several lines of evidence indicate that the hepatocytolytic activity of the immune system is mainly mediated by the adaptive immunity (for review see [18]). However, components of the innate immunity, namely NK and NKT, can actively participate in the pathogenetic mechanisms by killing infected cell and, less directly, by the production of chemokines and cytokines with antiviral and pro-inflammatory activity as well as by shaping the adaptive immune response [19]. In HCV infection, a strong inhibition of NK cell response has been documented [20] and the mechanisms of this impairment could be related to the effects of E2 protein on the CD81 molecule in NK cells [21].

#### Adaptive immune response

Concerning the adaptive immune response, it is well known that, in the acute phase of infection, a vigorous and multispecific T cell response is correlated with HCV clearance, whereas, in patients with chronic infection, the T cell response is generally delayed, transient and narrowly focused [22,23]. The dominant role of the adaptive immunity in determining the liver injury is confirmed by the detection of HCV-specific T lymphocytes in the peripheral blood or in the liver, several weeks after the infection and in coincidence with the peak of transaminase elevation, while no cytolytic activity is observed during the massive viral replication preceding this phase [24]. The depletion of cytotoxic CD8+ T lymphocytes (CTLs) at the peak of HCV viremia (and not of the helper CD4+ T cells) significantly delays the onset of the biochemical and clinical evidence of hepatitis [18]. Furthermore, the strong association between the magnitude of HCV-specific CTL response and the liver disease has been demonstrated both in chimpanzees and patients with acute and chronic HCV infection [25].

The analysis of mechanisms of HCV-mediated liver damage have been mainly focused on the role of CD8+ T lymphocytes due to the effector role of this cellular component. HCV-specific CD8+ cells exert their action in limiting viral infection by a dual mechanism: they contribute to the clearance of infected cells by inducing apoptosis through the release of cytotoxic granules of granzyme B that are internalized by the formation of perforin-induced pores in the hepatocyte membrane. Granzyme B cleaves pro-caspases which prompts the caspase cascade that leads to cell apoptosis. In addition, the activation of the Fas/Fas-ligand pathway leading to cytochrome C release and Caspase 8 activation has also been documented [26]. In fact, the overexpression of Fas molecules on HCV infected hepatocytes has been detected, as well as the expression of Fas-ligand on the surface of CD8+ T cells infiltrating the liver [27,28]. Besides their cytotoxic activity, CTLs contribute to the inhibition of viral replication by the release, after antigen recognition, of antiviral cytokines, mainly IFN-γ, as confirmed by the viral clearance in HCV-infected chimpanzees in the presence of IFN-γ secreting CTLs, without evidence of liver disease [29]. Although initiation of the cytopathic activity is clearly attributable to HCV-specific CTLs, it is difficult to explain the amplitude of liver cell destruction as only secondary to the elimination of HCV-infected cells. In fact, the number of apoptotic hepatocytes (7-20% according to the proportion of cells with caspase-3 activation) seems much higher than the fraction of infected liver cells (generally estimated in 1-10%) [30]. This discrepancy could be explained by the so-called “bystander killing” of hepatocytes not bearing HCV antigens. The number of HCV-specific CTLs present in the liver is outnumbered by recruited, HCV-nonspecific, T cells and other inflammatory cells and indeed this number may exceed 90% [31,32]. This additional cell population can contribute to the bystander activation. Even though the mechanisms of liver injury of antigen-nonspecific inflammatory cells are not fully defined, these include the production and secretion of proinflammatory cytokines, chemokines and cytotoxic mediators such as perforin, granzyme B, TNF-α, nitric oxide, etc. [18].

Besides the previously described role of CTLs, the CD4Besides the previously described role of CTLs, the CD4+ helper T lymphocytes play a significant role in the control of HCV infection and, less directly, in the consequent liver damage. The central role of helper T cells as regulators of the immune response includes the facilitation and maintenance of virus-specific CTL as well as B-lymphocyte function and antibody production. Several reports have shown that a HCV-specific CD4+ T cell response is necessary to activate an effective CTL response and to control viral infection [33] as also witnessed by the presence, in patients that resolved HCV infection, of a vigorous, multi-epitope specific, Th1 type and sustained CD4+ T cell response, constantly accompanied by strong CD8+ activation. Conversely, in chronic carriers, the CD4+ T cell response was weak, time limited and narrowly selected [34]. It seems unlikely that helper T lymphocytes play a major role in liver damage, even if a direct contribution to liver injury of this cellular component has been hypothesized. However, the magnitude of HCV specific CD4+ T cell response directly correlates with the rate of progression of chronic liver disease [35]. It is conceivable that the helper T cell population could contribute to the pathogenetic process with the secretion of soluble factors responsible for the recruitment of inflammatory cells in the liver and the consequent hepatocellular killing.

#### Humoral immunity

The role of the humoral immunity in the control of viral infection and in the pathogenesis of HCV-related liver damage is controversial. Naturally acquired HCV antibodies cannot protect from reinfection [36,37] and HCV infection can resolve without developing anti-HCV antibodies [38]. However, in the chimpanzee model, it was possible to neutralize HCV infectivity by *in vitro* treatment with antibodies taken from chronically infected HCV patients [39]. The existence of neutralizing antibodies was confirmed by several studies and the ineffectiveness in protecting from reinfection probably reflects the sensitization to virions that were counterselected and have been substituted by mutated viral species escaping the immune response. The possible contribution of antibodies, or even B cells, to liver damage is still being debated and indisputable results are lacking [40]. However, chronic HCV infection leads to autoimmune/lymphoproliferative disorders (see also below) and the tissue damage secondary to HCV-induced immune complexes has been largely documented (for review [41]).

#### Direct viral activity

Besides the previously described role of the immune system in the pathogenesis of HCV-related liver disease, a direct action of the virus has been proposed. HCV proteins (mainly core and NS5) have been shown to play a pathogenetic role in inducing oxidative stress in hepatocytes (for review see [42]). HCV core has been associated with the induction of lipid droplet accumulation and favoring of liver steatosis [43,44]. A direct oncogenetic effect of some HCV proteins has also been reported and may be involved in hepatocarcinogenesis. In fact, HCV core, NS3 and NS5A have been demonstrated, *in vitro*, to alter cell proliferation and apoptosis through different mechanisms including the activation of transcription factors, modulation of protoncogenes, inhibition of programmed cell death, interference with tumor suppressor proteins (for review see [45]).

### HCV and lymphoproliferative disorders

Early after its discovery, it was shown that HCV is also a lymphotropic virus [46]. As a consequence of the lymphatic infection, several lymphoproliferative disorders (LPDs) have been associated with HCV infection [47], including mixed cryoglobulinemia (MC) and B-cell non-Hodgkin's lymphoma (B-NHL) [48-59].

Mixed cryoglobulinemia, is a clinically benign, but pre-lymphomatous disorder, evolving in about 10% of cases, into a malignant lymphoma [41,47,60]. Therefore, it was hypothesized that HCV may be involved in the pathogenesis of B-NHL as well [46,49,54]. This hypothesis was substantiated by several observations including the significantly high prevalence of HCV infection in NHL patients, [51,52,54,58,59,61-65] - even with a higher prevalence in Southern countries - as well as the possible resolution of the disease following viral eradication [66]. In addition, in a recent study involving about 3,000 HCV-infected patients observed during a long-term follow-up, it was shown that the annual incidence of lymphoma was 0.23% and the cumulative rate of lymphoma development after 15 years was 2.6% in both the untreated and non-responder patients with persisting infection versus 0% in treated patients achieving viral eradication, strongly suggesting that the viral eradication protects against the development of lymphoma [67]. Several histopathological types of lymphoma have been observed in HCV patients, the most strictly associated being the lymphoplasmacytic, marginal zone and diffuse large B-cell lymphoma, as also shown in a recent very large multicenter study [68].

Interestingly, the de novo appearance or exacerbation of HCV-related LPDs have been shown in conditions of persisting immunosuppression, like the liver transplantation [69-71].

### Mechanisms of lymphomagenesis

#### Sustained antigenic stimulation

Several hypotheses have been proposed concerning the possible mechanisms of HCV lymphomagenesis. First, sustained antigenic stimulation has been suggested to play a key role in inducing the B-cell clonal expansion characterizing these disorders and it has been suggested that the same HCV antigens may be involved in the induction of both MC and lymphoma [72,73]. In other studies it has been suggested that HCV E2 and NS3 proteins represent the involved antigens. Particular attention was focused on the E2 protein. It has been shown that E2 interacts with the tetraspannin CD81, present also on the B-cell surface and it has been suggested that this binding is responsible for a sustained polyclonal B-cell activation essentially by lowering the B-cell activation threshold [74,75]. In addition, E2 protein has been suggested to be the inciting antigen of HCV-related NHL [76]. A specific expression of particular VH genes (VH1-69) in MC monoclonal B-cells has been demonstrated [77] and an accelerated apoptosis and marked anergy of these cell populations in MC patients have been recently reported [78,79]. Several studies have suggested that some cytokines, including IL-1, IL1 inhibitors and some chemokines, play a role [80-82]. Among these, particular attention has focused on B-cell activating factor (BAFF or BLyS). High levels of this cytokine were shown in patients with HCV-related autoimmune and/or LPDs and especially in MC [83]. We have investigated the reasons for such elevated levels and shown that MC patients were characterized by a higher prevalence of a particular allele of the gene promoter previously shown to be associated with enhanced transcriptional activity. Furthermore, T homozygosis was associated with significantly higher levels of the cytokine in the patients’ serum [84].

#### Viral lymphotropism

Conflicting data are available concerning the lymphatic infection in patients with LPDs, probably due to technical difficulties. However, some data are of interest, starting from the observation of a more evident infection of peripheral blood mononuclear cells (PBMC) in patients with MC than in patients without [49]. In a study using the model of injection of PBMC from HCV-positive patients into SCID mice, it was shown that the samples derived from HCV patients with malignant LPDs were characterized by positivity for HCV replicative intermediates, stronger signals when tested for HCV genomic sequences and successful serial passage of infected cells in different animals [85]. More recently, Sung and coworkers showed the establishment of B-cell lines persistently producing infectious virus from an HCV-positive lymphoma [86]. Finally, using a model of *in vitro* HCV infection of B-cells, it was possible to show that this infection may induce an enhanced mutation rate of immunoglobulins and some oncogenes, possibly through the induction of error-prone DNA polymerase and AID, suggesting that HCV may cause tumors by a hit and run mechanism [87]. More recently, Ito and coworkers observed a dramatically increased expression of AID in the B-cells of HCV patients, suggesting that this may represent a key lymphomagenetic factor [88].

#### Direct activity of viral proteins

In regard to viral proteins, particular attention has been focused on the HCV core protein due to previously shown pleiotropic effects on different cell signaling pathways modulating cell viability and proliferation [45]. Focusing on animal models, core transgenic mice developed lymphoma with high frequency [89]. In another transgenic model, the expression of the HCV core in the context of all structural proteins and in a irf −/− background, was associated with the development of lymphoid disorders including frank lymphoma [90]. More recently, the expression of the whole HCV genome in the B-cell compartment resulted in a high prevalence of diffuse large B-cell lymphoma [91]. Interestingly, the HCV core gene was expressed in all lymphomas. Finally, in a study performed on both B-cell lines expressing the HCV core protein and in primary B-cells from patients with LPDs, it was possible to show the altered expression of some isoforms of genes of the p53 family, the DNp63 and DNp73, previously shown to be overexpressed in human cancers, including lymphoma [92].

#### Chromosomal aberrations

Interesting data also exist about the possible role played by chromosomal aberrations, the most studied being the t(14;18) translocation. This translocation was found to be significantly associated with type II or monoclonal MC and the overexpression of the antiapoptotic *bcl2* gene in B-cells, resulting in an imbalance of the Bcl2/Bax ratio and abnormal B-cell survival [93-96]. The regression of the expanded B-cell clones following effective antiviral treatment and, in some relapsing patients, a new expansion of the same clones was also shown [97]. Finally, in a long-term follow-up study, an occult HCV persistence limited to the lymphatic compartment was observed in some patients resulting sustained virological responders after antiviral therapy [98,99]. More interestingly, such a persistent occult lymphatic infection was associated with the initial diagnosis of MC, the persistence of some MC symptoms after therapy and of expanded translocated B-cell clones. The occurrence, even if rare, of persisting MC disease, in spite of complete viral eradication, suggests the existence of points of no return [98,99]. The high prevalence of t(14;18) in HCV-related MC was shown by different authors using various methodological approaches including PCR-based methods, sequencing and fluorescence in situ hybridization with probes [93-96,100,101]. Contrasting data were also reported, probably due to different methodological approaches. Sansonno and coworkers could not show the same frequency of bcl-2 rearrangement by performing PCR assays on nucleic acids extracted from portal tract isolated with laser capture microdissection from liver biopsy sections of 16 HCV patients with and without extrahepatic B cell-related disorders [102].

In conclusion, available data suggest that HCV lymphomagenesis is a complex multistep multifactorial process, probably based on sustained B-cell activation and the inhibition of B-cell apoptosis on a background of genetic predisposing factors and evolving through the progressive addition of genetic aberrations which allow the process to be progressively less dependent on the etiologic agent.

Overall, the pathogenesis of HCV-related hepatic and extrahepatic disorders (the so-called “HCV disease”) is still not completely known. In particular, the relationship between this infection and the immune system appears very complex and multifaceted. In this light, the analysis of the “in vivo” effects of a condition of general or variably selective impairment of the host’s immune response should evidence very interesting models of study, possibly helping to clarify still unclear pathogenetic mechanisms with high translational potentiality in the clinical approach to this complex condition.

The following sections will focus on the main available data concerning some conditions which are the principal source of information on the effects of immunosuppression in the presence of HCV infection, and which are often interlinked: liver or kidney transplantation, the use of some biologic drugs and cancer chemotherapy.

### HCV infection and transplantation

#### Liver transplantation

The field of liver transplantation (LT) has been well investigated and provides ample data about the effect of immunosuppression on HCV-related disease [103], even if the liver transplanted patient does not appear an optimal model. This appears secondary to the variety of protocols used in different studies and the many variables involved, accounting for the non-uniformity in conclusions from different studies.

A common observation is that, in the case of LT for HCV-related disease, reinfection of the graft is almost immediate and universal, and the progression of liver damage is five- to ten-fold faster compared to non-transplanted patients, so that up to 40% of patients experience recurrent hepatitis and cirrhosis 5 years later [104]. The accelerated course of post-transplant hepatitis C translates into a significantly higher rate of graft loss [105]. In addition, liver transplantation appears to be relevant also with reference to HCV LPD pathogenesis. As an example, de novo appearance or exacerbation of MC has been reported after LT, even if the involved mechanisms are still unclear [69-71].

The main differences consist in the interpretation of the role played by immunosuppressive drugs -especially corticosteroids (CS) and cyclosporin A (CsA) - used in the post-LT period, with different suggested therapeutic protocols.

An explanation for the hypothesis of different effects on recurrent HCV-related liver damage after LT using CS - or CsA - based protocols, can be found in recent studies performed *in vitro* using the replicon system and showing that - unlike what is known in the case of HBV - CS does not act by increasing viral replication, but by dramatically increasing the ability of HCV to enter into target cells and thus spread the infection through the transactivation and consequent overexpression of genes codifying for HCV cellular receptors (occludin, SR-B1) [106,107]. A recent report evaluating the safety and efficacy of steroid-free immunosuppression, showed that this approach is safe and effective for liver transplant recipients with chronic HCV, however, steroid sparing has no clear advantage in comparison with traditional immunosuppressive protocols [108]. Several studies have reported the antiviral effect of CsA. In fact, HCV is critically dependent on cyclofilin B to complete its intracellular replication. Consequently, the binding of cyclosporin A with cyclofilin B leads to the suppression of HCV replication. This suggests a clinical advantage in using CsA instead of tacrolimus (TAC) [109]. Both drugs are inhibitors of calcineurin (CNIs), but they act through a different mechanism since TAC acts through FKBP12 (for review see [110,111]) and does not have any antiviral effect [106].

There are numerous clinical studies reporting conflicting outcomes with each of the different immunosuppressive drugs used in the post-transplant period which condition the rapidity of liver damage progression. In spite of varying conclusions from different studies regarding the effect of CS avoidance on recurrent hepatitis C, a meta-analysis of the available randomized trials indicates that the relative risk of HCV recurrence reached statistical significance (*p* = 0.03) for a better outcome with CS avoidance [112]. In addition, there is general agreement about including treated episodes of acute cellular rejection and pulse therapy with CS, as well as longer duration/higher cumulative exposure to CS, among the factors which highly influence the negative impact of liver transplantation in HCV + patients [113-115]. These data suggest that the benefit of CS avoidance may be real, even if small. Because CS is not required for successful LT and its use is associated with several side effects, it has been suggested that CS minimization or avoidance would be an important practice in HCV patients [116].

Conflicting results are also reported for the use of CsA instead of TAC. In most retrospective studies, no difference was apparent [117-120]. In a meta-analysis, statistically significant differences between TAC-based vs. CsA-based therapies were not found for mortality, graft survival and fibrosing cholestatic hepatitis [121]. In a more recent prospective, randomized trial, no differences were found between the 136 patients allocated to CsA and the 117 on TAC [122].

A recent report, based on data received from the United Network for Organ Sharing, describing a very large cohort of 8809 chronic HCV liver transplant recipients, showed an increased risk of patient death and graft failure in CsA treated patients compared to TAC treated patients, suggesting to reconsider the targeted administration of CsA to HCV-infected liver transplant recipients [123]. However, in a multicenter study, the mean time to histological diagnosis of hepatitis C recurrence was significantly longer with CsA [124].

A beneficial impact of Sirolimus (RIR) on HCV recurrence was also suggested [125]. Interestingly, Sirolimus is becoming a relevant player in iatrogenic immunosuppressed patients to avoid HHV8 reactivation with consequent high risk of Kaposi sarcoma incidence. However, these data require further confirmation [126]. On the whole, immunosuppression determines HCV-related disease progression; however the effects of different immunosuppressive drugs used after transplantation (such as CNIs, CS, mycophenolate mofetyl - MMF -, azathioprine) on HCV recurrence are still equivocal and there are no convincing data to modify the currently used type of immunosuppression. At present, the only form of immunosuppression which has been undoubtedly and consistently associated with more severe hepatitis C recurrence is the treatment of acute cellular rejection, a condition that is typically treated with pulse CS or biological drugs (OKT3, thymoglobulin) [104,114,127]. Consequently, it is recommended to avoid steroid boluses. Furthermore, since insulin resistance and diabetes are associated with fibrosis in HCV-infected liver recipients, the use of immunosuppressive agents without this side effect may slow post-LT disease progression [128,129]. Prospective controlled studies aimed at definitely resolving these still unresolved questions are ongoing.

#### Kidney transplantation

Similar considerations may be translated into the less documented field of kidney transplantation in HCV-positive patients. Whereas a series of past studies including short-term follow-up post-transplantation suggested that there was no significant difference between HCV-positive or -negative patients, the existence of such a difference was shown to become significant after 5 years post-transplantation in studies including longer follow-up [130]. In the study by Fabrizi et al. [131] the meta-analysis of several observational studies showed that the survival of the transplanted organ was lower in HCV-positive patients and that the higher mortality rate was related to the increase in viral replication/liver damage.

### HCV infection and biological drugs

The effects of biological drugs on HCV infection and its sequelae are particularly interesting. A consistent amount of data exists about the anti-TNF agents (i.e., etanercept, infliximab, adalimumab). In a recent review by Ferri *et al.*, anti-TNF drugs result in being effective and well tolerated in the case of HCV-positive patients. The possible positive effect exerted by the inhibition of TNF-α (reputed to play a key role in the pathogenesis of HCV-related liver damage) is outlined in some studies and the possible efficacy of combined therapies, including both anti-TNF-α and standard anti-HCV treatment, has been suggested [132].

Of the biological drugs used in the treatment of HCV-positive patients, increasing interest during the last decade has been focused on the anti-CD20 monoclonal antibody rituximab (RTX), a B-cell specific immunosuppressant acting through transient depletion of the B-cell compartment. The use of RTX, initially confined to the onco-hematological area, has been progressively expanded to involve a growing number of autoimmune and benign B-cell lymphoproliferative conditions. Due to the etiopathogenetic role played by HCV in several autoimmune and/or LPDs, such as MC (see previous paragraphs), the effects of RTX in HCV-positive patients are of special interest. Patients undergoing RTX therapy for HCV-related MC appear to be a unique model of study. Beginning with pioneering studies in 2002–2003 [133-135], RTX has been shown to be efficacious in the treatment of the majority of MC symptoms and valuable in patients in whom antiviral therapy was contraindicated [40,132]. However, the observation of especially severe hepatitis reactivations after RTX use in HBV-positive patients, has justified the exclusion of also HCV-positive MC patients with advanced liver disease also. However, in two successive studies, it was possible to observe that RTX was useful and safe in MC patients with HCV-related advanced liver disease [40,136]. Interestingly, in these patients the treatment induced an unexpected, paradoxical positive effect on the liver disease. This was especially evident in cirrhotic patients with ascitic decompensation who experienced a consistent improvement of cirrhotic syndrome, including the disappearance of the ascites in some cases, improvement of protido-synthetic activity of the liver with increasing levels of plasmatic albumin, and a reduction of the Child-Pugh score. Viremia titers transiently increased and hepatocytolysis followed the progressive reconstitution of the B-cell compartment. On the whole, the average level of ALT did not increase [134]. These effects of RTX therapy [40], and the rapidity of their appearance following B-cell depletion, strongly suggested a consistent role played by modifications in the cytokine network and a previously unknown key role played by B-cells in the pathogenesis of HCV-related liver damage [40].

Apart from the consequences of the use of specific biological drugs, it is generally agreed that the risk related to a reactivation of hepatitis C in patients with autoimmune/rheumatological conditions treated with current immunosuppressive drugs, is of a consistently lesser extent than in the case of hepatitis B and generally associated with the use of combinations of different immunosuppressant agents.

### HCV infection and chemotherapy

Another complex issue involves the effect of HCV infection on patients undergoing chemotherapy. It is well known that in the case of patients with HBV infection, the most critical condition is represented by hematologic malignancies. In this field, some past studies have strongly suggested that chemotherapy generally does not have dramatic consequences: it is possible that liver damage occurs, but severe consequences have to be considered as rare events [137]. More recently, the study by Mailliard and coworkers offered a unique possibility to evaluate the impact of chemotherapy on oncology patients [138]. This study was based on the analysis of the long-term follow-up of victims of a nosocomial epidemic by the same strain of HCV G3a, in patients with either hematological or solid-tumor cancer undergoing chemotherapy. In this study, 100% of cases become chronic and developed severe liver damage (cirrhosis) over a very limited time, with significant mortality.

Recently, a review of the available literature revealed that, in the case of patients with hematological malignancies, the presence of HCV infection is associated with increased risk for sinusoidal obstruction syndrome (SOS), graft versus host disease (GVHD) and liver failure, but does not affect short-term survival in bone marrow transplant (BMT) recipients. So, infection with HCV in donor or recipient should not be considered an absolute contraindication for BMT [139].

More recently, several case reports or retrospective studies have outlined the potential risk of hepatitis C reactivation following regimens including chemotherapy and RTX in HCV-positive lymphoma patients. When compared with the above cited effects of RTX monotherapy on a HCV-related benign lymphoproliferative disorder (MC) these recent data suggest more serious effects on viral replication and ALT flares in oncohematological patients [140-143]. Despite the paucity of available data as well as the existence of some more reassuring results from different studies, it seems conceivable that situations involving both a baseline immunosuppressive status - linked to the oncohematological condition - and a combination of different immunosuppressive drugs, may result in a varying, more aggressive evolution of liver damage. While waiting for more consistent data, a recent meta-analysis of available studies concludes that even in such situations, the presence of infection should not be considered a contraindication to the use of therapies based on RTX, especially in patients without initial liver dysfunction.

Overall, the current data appear insufficient to draw definitive conclusions regarding the effect of HCV viral load, reactivation, and treatment on the prognosis of cancer, and especially in patients with lymphoma.

## Conclusions

Liver disease in an immunosuppressed patient is typically severe with an unusually rapid progression to cirrhosis. However, the combination of HCV infection and immunosuppression may lead to different conditions ranging from enhancement to inhibition of HCV replication/infection and from worsening to improvement of liver damage. These possibilities should be accurately evaluated in each patient, taking into consideration variables such as the type of immunosuppression and the baseline liver damage/pathological condition to be treated. These characteristics make the study of this situation very promising in the field of immunopathogenesis of both hepatic and extrahepatic HCV-related diseases.

From a practical point of view, the risk of developing rapidly evolving, difficult-to-control liver disease appears to be lower than in the case of HBV infection and so a less cautious approach is allowed if immunosuppressive therapy is considered urgent and necessary. However, accurate screening and specialized advice is recommended as soon as possible in HCV-positive patients.

## Competing interests

All authors declare the absence of any conflict of interest.

## Authors' contributions

ALZ, drafted and wrote the manuscript; CG, LG, AP and EF contributed to the writing of the paper and performed the bibliographic research. All authors read and approved the final manuscript.
